# Supplementary material for: Fast and memory-efficient mapping of short bisulfite sequencing reads using a two-letter alphabet
Source: NAR Genom Bioinform. 2021 Dec 22;3(4):lqab115. doi: 10.1093/nargab/lqab115 (PMC8693577; doi:10.1093/nargab/lqab115)
Supplement: lqab115_Supplemental_Files [file lqab115_supplemental_files.zip › supplementary_scripts.zip!/abismal_benchmark/results/figures/accuracy.pdf]

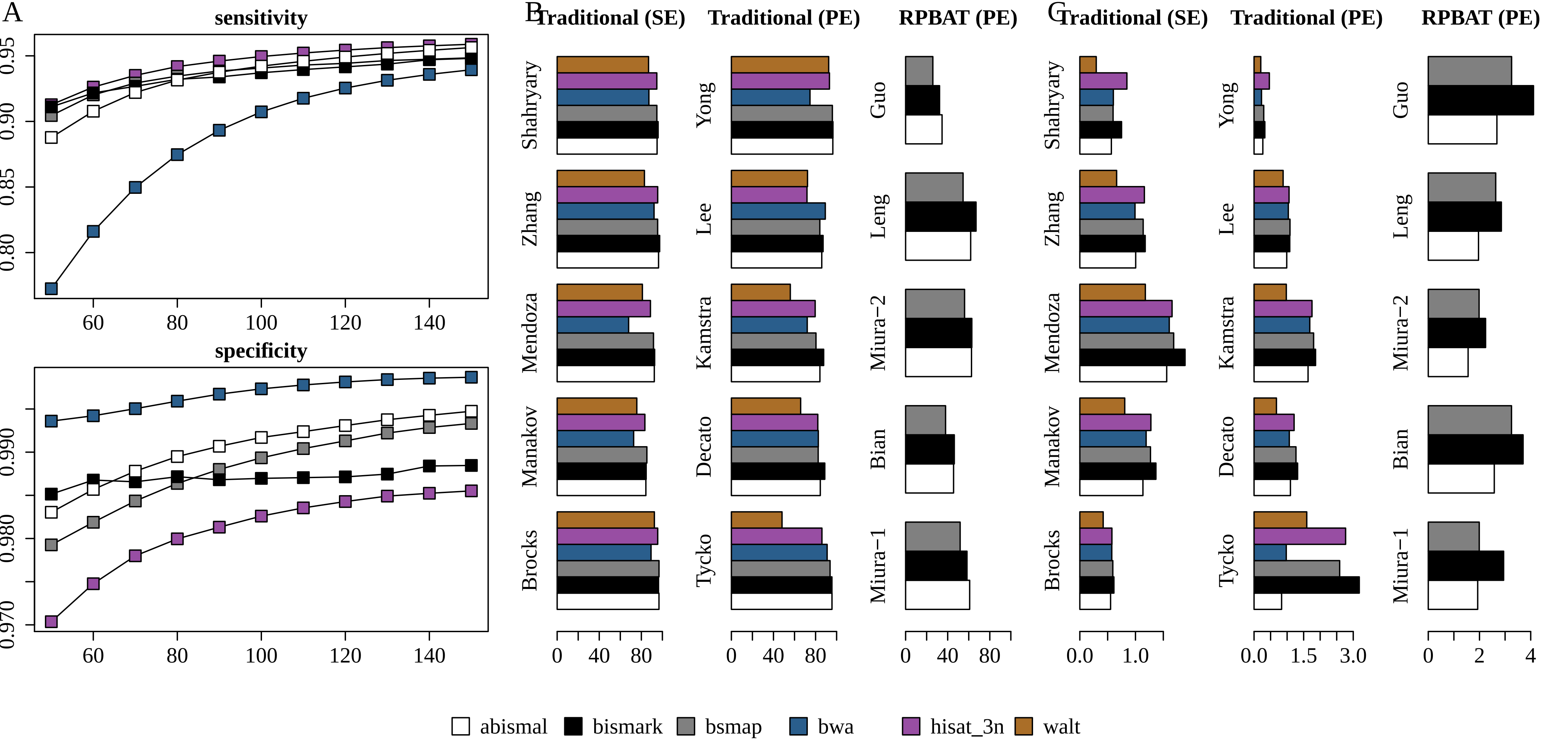

A
sensitivity
specificity
B
C
Traditional (SE)
Traditional (PE)
RPBAT (PE)
Shahryary
Zhang
Mendoza
Manakov
Brocks
Yong
Lee
Kamstra
Decato
Tycko
Guo
Leng
Miura−2
Bian
Miura−1
abismal
bismark
bsmap
bwa
hisat_3n
walt
