# Supplementary material for: Fast and memory-efficient mapping of short bisulfite sequencing reads using a two-letter alphabet
Source: NAR Genom Bioinform. 2021 Dec 22;3(4):lqab115. doi: 10.1093/nargab/lqab115 (PMC8693577; doi:10.1093/nargab/lqab115)
Supplement: lqab115_Supplemental_Files [file lqab115_supplemental_files.zip › supplementary_scripts.zip!/abismal_benchmark/results/figures/supp_2_simulations_rpbat.pdf]

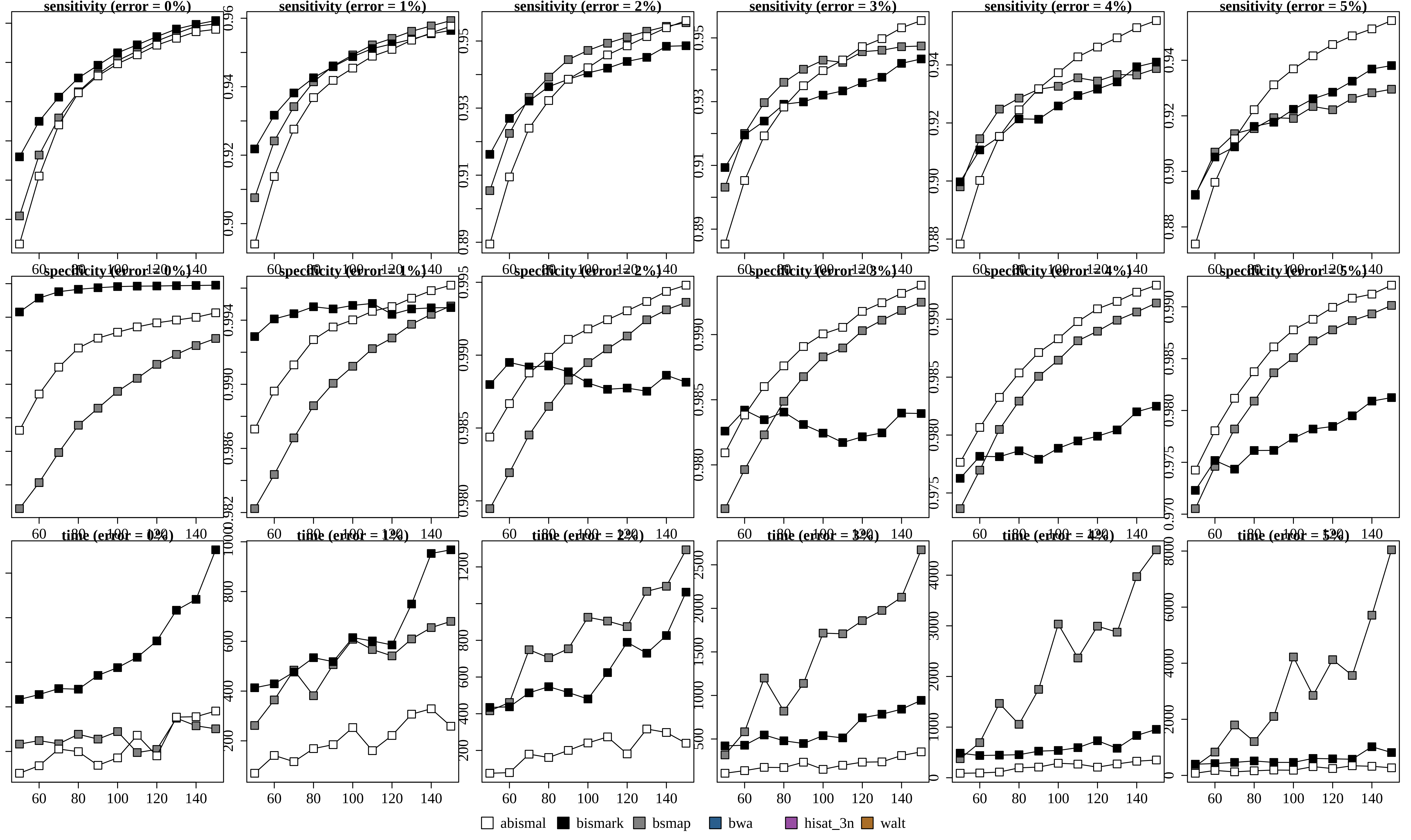
sensitivity (error = 0%)
sensitivity (error = 1%)
sensitivity (error = 2%)
sensitivity (error = 3%)
sensitivity (error = 4%)
sensitivity (error = 5%)
specificity (error = 0%)
specificity (error = 1%)
specificity (error = 2%)
specificity (error = 3%)
specificity (error = 4%)
specificity (error = 5%)
time (error = 0%)
time (error = 1%)
time (error = 2%)
time (error = 3%)
time (error = 4%)
time (error = 5%)
abismal
bismark
bsmap
bwa
hisat_3n
walt
