# Supplementary material for: Fast and memory-efficient mapping of short bisulfite sequencing reads using a two-letter alphabet
Source: NAR Genom Bioinform. 2021 Dec 22;3(4):lqab115. doi: 10.1093/nargab/lqab115 (PMC8693577; doi:10.1093/nargab/lqab115)
Supplement: lqab115_Supplemental_Files [file lqab115_supplemental_files.zip › supplementary_scripts.zip!/abismal_benchmark/results/figures/resources.pdf]

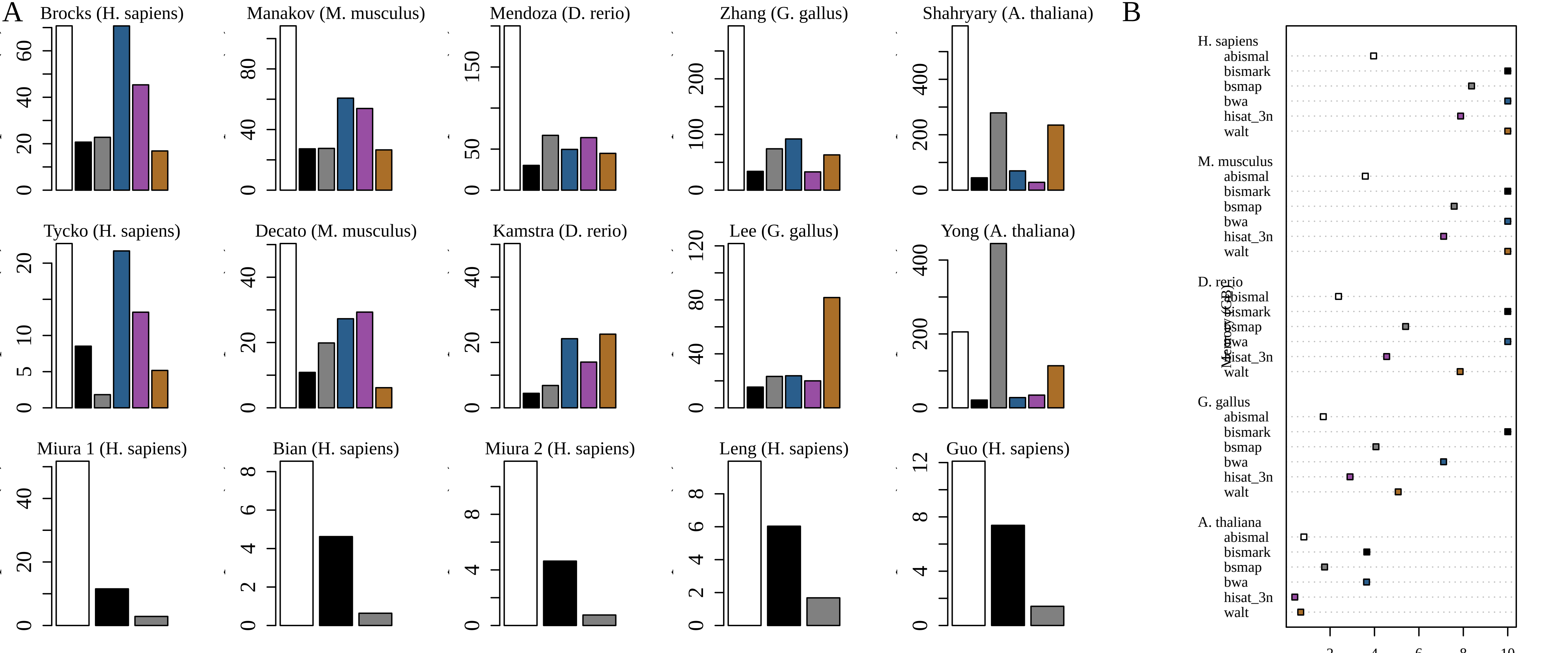

A
Brocks (H. sapiens)
Manakov (M. musculus)
Mendoza (D. rerio)
Zhang (G. gallus)
Shahryary (A. thaliana)
Tycko (H. sapiens)
Decato (M. musculus)
Kamstra (D. rerio)
Lee (G. gallus)
Yong (A. thaliana)
Miura 1 (H. sapiens)
Bian (H. sapiens)
Miura 2 (H. sapiens)
Leng (H. sapiens)
Guo (H. sapiens)
B
Memory (GB)
H. sapiens
abismal
bismark
bsmap
bwa
hisat_3n
walt
M. musculus
abismal
bismark
bsmap
bwa
hisat_3n
walt
D. rerio
abismal
bismark
bsmap
bwa
hisat_3n
walt
G. gallus
abismal
bismark
bsmap
bwa
hisat_3n
walt
A. thaliana
abismal
bismark
bsmap
bwa
hisat_3n
walt
