# Supplementary material for: Fast and memory-efficient mapping of short bisulfite sequencing reads using a two-letter alphabet
Source: NAR Genom Bioinform. 2021 Dec 22;3(4):lqab115. doi: 10.1093/nargab/lqab115 (PMC8693577; doi:10.1093/nargab/lqab115)
Supplement: lqab115_Supplemental_Files [file lqab115_supplemental_files.zip › supplementary_scripts.zip!/abismal_benchmark/results/figures/fig1b_collision.pdf]

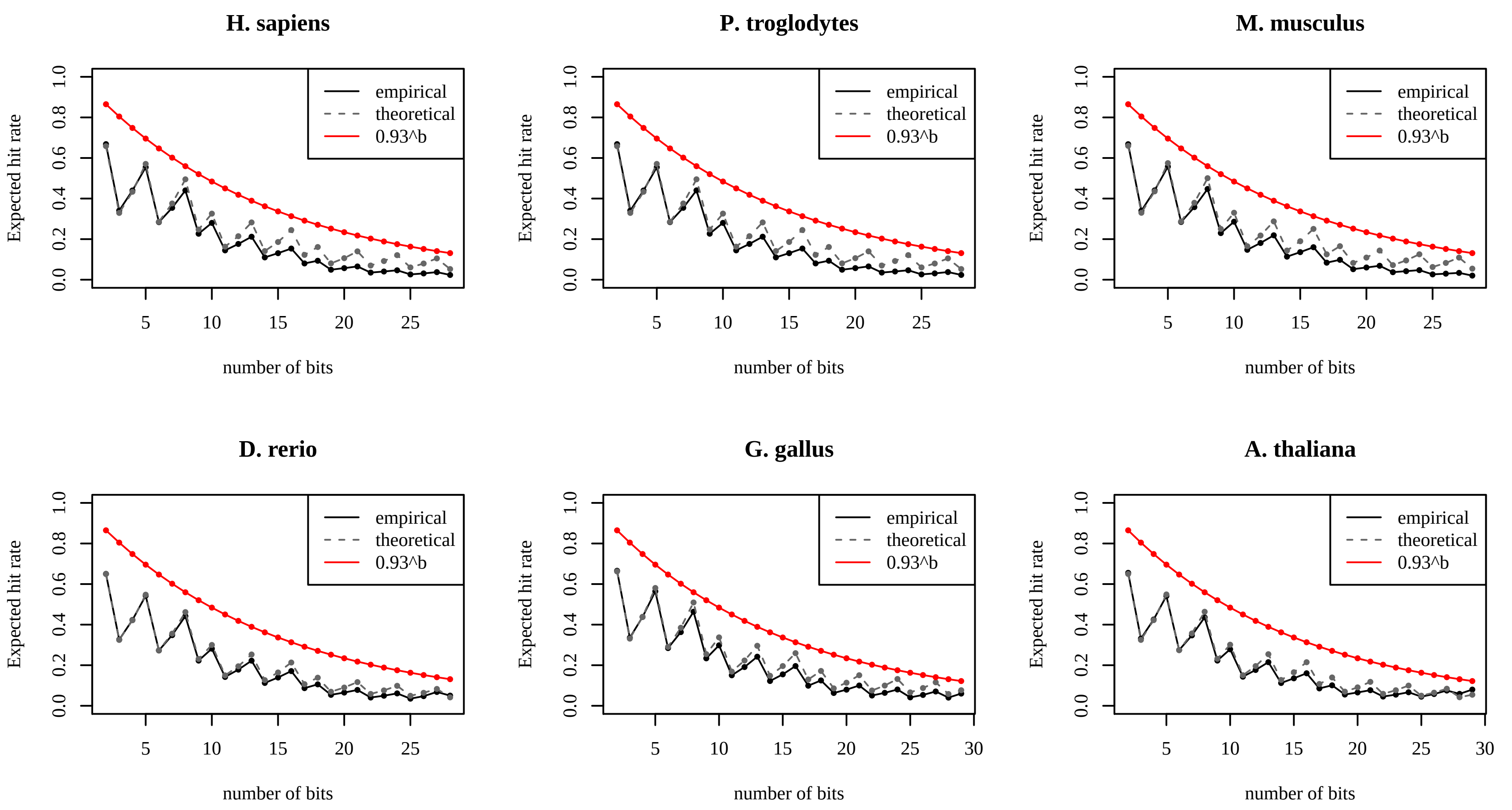

H. sapiens
P. troglodytes
M. musculus
D. rerio
G. gallus
A. thaliana
empirical
theoretical
0.93^b
Expected hit rate
number of bits
0.0
0.2
0.4
0.6
0.8
1.0
5
10
15
20
25
30
